# Enhanced Electrochemiluminescence by Knocking Out Gold Active Sites

**DOI:** 10.1002/anie.202421185

**Published:** 2024-12-27

**Authors:** Indhu Leka Kottaiveedu Sivakumar, Laurent Bouffier, Neso Sojic, Shanmugam Senthil Kumar

**Affiliations:** ^1^ Electrodics and Electrocatalysis Division CSIR-Central Electrochemical Research Institute (CSIR-CECRI) Karaikudi Tamil Nadu 630003 India; ^2^ Academy of Scientific and Innovation Research (AcSIR) Ghaziabad 201002 India; ^3^ University of Bordeaux CNRS Bordeaux INP ISM UMR, 5255 F-33400 Talence France

**Keywords:** Electrochemiluminescence, Cu-Fenton oxidation processes, Mechanism, Active sites, Gold surface

## Abstract

Electrochemiluminescence (ECL) of the conventional system of [Ru(bpy)_3_]^2+^ luminophore and amine‐based coreactants is particularly inefficient on noble metal electrodes. This is due to the formation of a passivating oxide layer on the metal surface inhibiting the electro‐oxidation of amines like tri‐*n*‐propylamine (TPrA) coreactant. Herein, we demonstrated the enhancement of ECL emission on gold surface by hydroxyl radicals attack that are chemically generated with Cu‐Fenton reagent. These radicals selectively deactivate the gold active sites and knockout the metal surface asperities that counterintuitively led to an amplification of the ECL emission. Atomic force microscopy shows a massive smoothening of the surface. The electrochemical characterization proves that the involved ECL reaction mechanism switches from direct oxidation to catalytic route, where the kinetics of indirect TPrA oxidation is facilitated on deactivated gold surface. Besides, in situ smoothening of a rough electrode in presence of tandem [Ru(bpy)_3_]^2+^/TPrA enables Cu^2+^ sensing with good reliability and limit of detection. Such atomically smoothened and corrosion‐resistant gold surface readily tuned the ECL reactivity and opened new directions on influence of topography and reactivity on ECL mechanisms, thus will be extremely useful for the future development of ECL imaging strategies and highly sensitive ECL sensors.

## Graphical abstract

Passivating oxide formation in ideal gold electrodes reflects in weaker ECL intensity of conventional [Ru(bpy)_3_]^2+^/TPrA systems. Such hampering behavior were eliminated by selective knocking out of surface asperities and catalytic deactivation via Cu‐Fenton reagent. Results exhibited 2.7‐fold increase in ECL intensity with switching mechanism from direct oxidation to catalytic pathway.

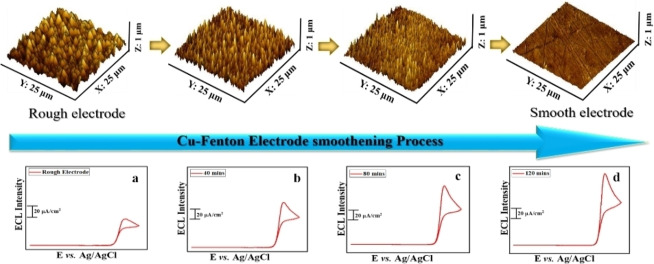

## Introduction

Gold is a primary solid metal electrode with great properties such as high conductivity, chemical inertness and good resistance to surface oxidation in water at mild positive applied potentials.^[1]^ It is utilized as an electrode material in fuel cells, optical sensors, and to promote various redox reactions.[^2^, ^3^, ^4^, ^5^, ^6^, ^7^] Furthermore, the chemical nature and topography of gold surface are of utmost importance to achieve analytical applications regarding electrochemical analysis, electrical contact quality, light scattering, surface‐enhanced Raman spectroscopy (SERS), plasmonics and direct self‐assembly of objects.[^8^, ^9^, ^10^, ^11^, ^12^, ^13^, ^14^] A macroscopic gold surface is composed of equilibrated metal surface (EMS) and metastable metal surface (MMS) or asperities. Among them, disordered, low coverage, high energy, electropositive, protruding asperities with a low lattice coordination number are responsible for catalyzing numerous electrochemical reactions.^[15]^ However, smooth and deactivated surfaces without asperities, showing high surface reflectivity are required for good quality spectral signals, well‐tailored Raman peaks, sharp and intense surface plasmon resonance, and are used as well in microelectronics.[^16^, ^17^, ^18^, ^19^] Also, atomically smooth gold electrodes serve as an efficient platform for constructing highly ordered molecular surfaces and low‐defect self‐assembled monolayers due to their lack of grain boundaries and step edges,^[20]^ and prevent unwanted corrosive reactions.^[21]^ In the early 2010s, Scholz and co‐workers demonstrated the selective knockout of such asperities using hydroxyl radicals (HO^**•**^) generated by Fenton reagent. In that case, the removal of gold active sites caused a progressive shift of the oxygen reduction reaction (ORR) peak towards more negative potential values.^[21]^ Later, they demonstrated the selective removal of surface asperities by the HO^**•**^ produced from an electro‐Fenton reagent. They further verified the smoothening effect by the decreased Pb underpotential deposition charge due to a diminished surface area of *circa* 30 %.^[8]^ Finally, they showed that the decrease of the catalytic activity of gold following this Fenton treatment was related to the removal of the surface atomic defects made of partially filled *d* orbitals that interact with the free radical intermediates involved in electrochemical reactions. Jo et al. reported similar two‐fold smoothening of a gold surface by ultrasonic irradiation and the role of HO^•^ in asperities removal by UV absorption spectroscopy and atomic force microscopy (AFM) imaging.^[22]^ Karthick et al. studied the smoothening of a gold electrode by the removal of Prussian blue/gold nanocomposite deposits.^[23]^ All these prior set of experiments are self‐consistent and proved that asperities removal leads to atomic‐level smoothening, corrosion resistance, catalytic deactivation and slow kinetics of inner‐sphere electron‐transfer (ET) reactions involving free radical intermediates.

As reported previously,[^23^, ^24^, ^25^] unlike a perfect crystallographic gold surface with completely filled *d*‐orbitals, the low‐coordination, protruding surface adatoms or asperities show a partially unfilled d^10−x^ (with *x*=1) electronic configuration. These *d*‐orbitals makes them prone to oxidation and responsible for their enhanced electrocatalytic activity, especially to the reactions involving free‐radical intermediates.^[25]^ Removal of such active site atoms by hydroxyl radical attack leads to a reduced electrocatalytic activity, which might indeed affect the capability to trigger electrochemiluminescence (ECL). ECL is a light‐emitting process based on the excitation of a luminophore triggered by exergonic ET reactions at the electrode/electrolyte interface.^[26]^ Its simplicity, feasibility, sensitivity, selectivity, high spatial and temporal control, versatility and low background noise make ECL an extraordinary readout in the field of analytical methods and imaging technologies.[^26^, ^27^, ^28^, ^29^] Among various ECL systems, a highly efficient one operating at physiological pH is composed of [Ru(bpy)_3_]^2+^ luminophore with tri‐*n*‐propylamine (TPrA) sacrificial coreactant. It is widely used for various bioanalytical and microscopy applications, from immunoassays to cell bio‐imaging.[^30^, ^31^, ^32^, ^33^, ^34^, ^35^, ^36^] Previous works reported the crucial role of TPrA oxidation for heterogeneous ECL‐based immunoassays and for ECL microscopy of biological entities.[^37^, ^38^, ^39^, ^40^] Nonetheless, this model system shows a declined ECL efficiency on gold as well as on other noble metal or carbon electrodes due to their surface oxidation occurring in the same potential region than that necessary for TPrA oxidation.[^41^, ^42^, ^43^, ^44^] In aqueous media, which is required for most bioanalytical and bio‐imaging experiments, a major drawback is the formation of surface oxides that inhibit the electro‐oxidation of TPrA.[^41^, ^42^, ^45^] This issue was partially overcome by modifying electrode surface with surfactants or alkanethiols to increase hydrophobicity and inhibit oxide formation.^[44]^ But, once the electrode is modified with a molecular layer, the stability of the functionalization remains an issue at the high anodic potentials required to generate ECL.[^46^, ^47^, ^48^, ^49^, ^50^]

In this work, we tuned the electrochemical reactivity of the gold surface to promote ECL by the selective removal of asperities and deactivation of the active gold sites using HO^•^ generated from copper Fenton (Cu‐Fenton) reagent. The standard ECL system of [Ru(bpy)_3_]^2+^/TPrA was used to quantify the asperities removal from an initially rough gold electrode surface. We studied in details the effects of Cu‐Fenton treatment on the electrode surface and its consequences on ECL emission, revealing a substantial increase of the signal intensity by switching from a direct oxidation mechanism to a catalytic oxidation mechanism. Our work opens exciting opportunities for ECL on different surface reactivity and topography.

## Results and Discussion

**Figure 1:**
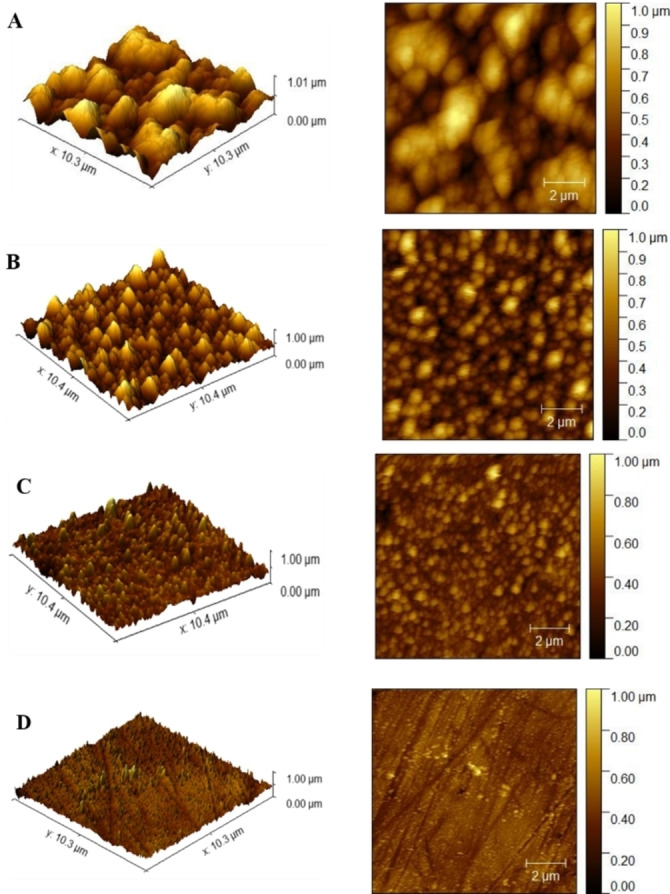
Topographic 3D (left) and top view (right) AFM images of a roughened Au electrode before (A) and after exposure to HO^•^ radicals for 40 (B), 80 (C) and 120 minutes (D) via ex situ Cu‐Fenton treatment.

To study a wide variety of surface roughness and distribution of asperities on the Au surface, the initially polycrystalline (Pc) electrodes were first roughened electrochemically. For that, the potential of the electrode was pulsed from 0 V to 2 V vs. Ag/AgCl for 30 s (i.e. 750 cycles of 20 ms‐long pulses). The topography of the gold surface was then investigated by AFM imaging after the electrochemical treatment. As expected, we observed that the Au surface appeared substantially rougher after this electrochemical process (Figure 1A). The AFM images of the gold Pc surface before and after ex situ Cu‐Fenton treatments are presented on Figure 1. The roughened electrodes were then exposed to a freshly prepared Cu‐Fenton reagent containing 10 mM CuSO_4_ and 100 mM H_2_O_2_ (i.e. ex situ Cu‐Fenton treatment). As shown in earlier studies, the HO^**•**^ generated are capable of effective smoothening of gold surfaces^[8]^ and of selective knocking out of asperities involved in electrocatalysis.^[21]^ However Cu‐Fenton reagent was selected instead of Fe‐based Fenton one in order to avoid the drawbacks of a slow reduction rate of Fe^3+^ cations, a sludge generation of reactive oxygen species and a limited working pH range.^[51]^ The mechanism producing the HO^**•**^ with the Cu‐Fenton reagent involves several advanced oxidation steps and is described in the Supporting Information (eqs. S1–S5). The produced HO^•^ radicals selectively knocked out gold atoms from the active surface and thus attacked the asperities of gold. The effects of this Fenton‐type “polishing” is illustrated by the resulting AFM images that display a progressive decrease of the roughness with longer exposure times (40 to 120 minutes) to the ex situ Cu‐Fenton reagent (Figures 1A−D). To perform a more quantitative analysis, the root‐mean‐squared roughness (*R_RMS_*) was calculated from each of these AFM images (equations S6–S8). The *R_RMS_* value for the Pc gold electrode increased from 40±8 nm (Figure S1A) to 120±20 nm (Figure 1A) after the chronoamperometric pre‐treatment. It demonstrates without ambiguity the effective roughening of the electrode surface using such an electrochemical procedure.

The top‐view topography and 3D AFM images of the chronoamperometrically roughened and ex situ smoothened electrodes at varying time intervals are shown in Figures 1B−D. The initially roughened gold electrode exhibits a progressive decrease in *R_RMS_* values from 120±20 nm to 53.5±1.9 nm, 25.4±1.2 nm and 16.2±0.4 nm after 40, 80 and 120 minutes of Cu‐Fenton treatment, respectively. It demonstrates that the electrode surface was efficiently smoothened by the removal of active gold atoms and asperities after the ex situ treatment of the electrode with Cu‐Fenton reagent. The decrease in *R_RMS_* value from 120 nm down to 16.2 nm was complemented by a decrease of the electrochemically active surface area from 0.155 cm^2^ to 0.077 cm^2^ as well as the roughness factor value from 2.2 to 1.09, a value of 1 being an ideally polished Pc−Au surface (Figure S2).

The electrochemical properties of the gold surfaces prepared with different roughness values were studied by cyclic voltammetry using two redox probes. Figure S3 shows the cyclic voltammograms (CVs) of K_3_[Fe(CN)_6_] recorded on a gold Pc electrode before and after immersion in Cu‐Fenton reagent for 60 minutes. The one‐electron ET reaction associated with [Fe(CN)_6_]^3−^/[Fe(CN)_6_]^4−^ occurs via an outer‐sphere mechanism without the involvement of any free radical intermediate. No change in peak current, peak potential and peak separation (*▵E_p_*) values were observed indicating the unaltered ET rate of this outer‐sphere process (Figure S3). However, large differences in *▵E_p_*, peak potential and peak current values were observed in the corresponding CVs when using hydroquinone (H_2_Q) as a redox probe (Figure S4). H_2_Q oxidation to form quinone (Q) involves a 2‐electron transfer coupled with protons transfer so that an inner‐sphere mechanism involving a free radical intermediate does occur. Therefore, the kinetics of this ET reaction is highly sensitive to localized environment.^[52]^ In other words, when the asperities that stabilize free radical intermediates are removed from the electrode surface, Q/H_2_Q electrochemical reactions become slower until their ET pair of peaks appear irreversible at a given scan rate. This is illustrated in Figure S4A by a sharp decrease in anodic and cathodic peak currents before and after the HO^**•**^ attack on the gold electrode. These results are indeed consistent with previous reports.[^21^, ^22^]

**Figure 2:**
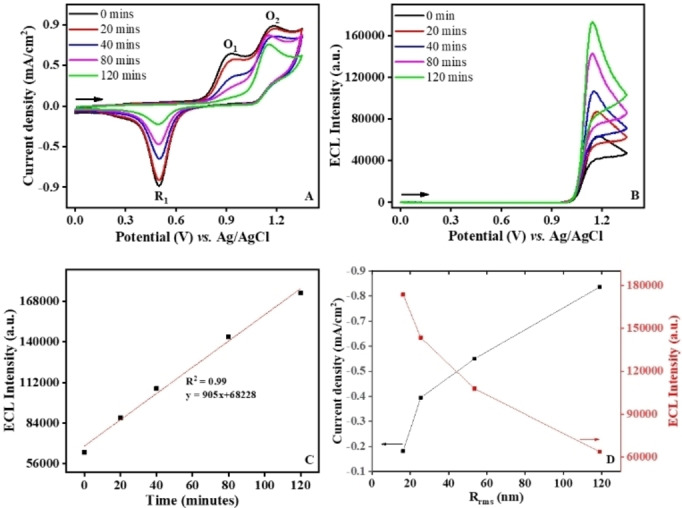
CV (A) and corresponding ECL (B) signal of 10 mM TPrA and 1 mM [Ru(bpy)_3_]^2+^ in 0.1 M PBS (pH 7.4) before and after different exposure times of Au electrode to HO^**•**^ by Cu‐Fenton reagent. Plot of the ECL intensity in function of the ex situ Fenton treatment duration (C). Variations of the gold reduction current density measured at 0.48 V and ECL with the surface roughness (D).

To study the ECL behavior, the effect of the HO^**•**^ attack on gold was then investigated by recording simultaneously the current and the luminescence intensity during CVs in solutions containing the ECL reagents. In Figure 2A, a small shoulder appears first at 0.3 V before two successive irreversible oxidation waves O_1_ and O_2_ as well as a single reduction process R_1_. The shoulder at 0.3 V was clearly visible at higher concentrations of TPrA (Figures S5 and S6) and it increased with the coreactant concentration. Therefore, the reaction occurring at 0.3 V can be ascribed to the catalytic oxidation of TPrA by hydrous oxide species formed by pre‐monolayer oxidation of active sites on the gold surface.[^41^, ^42^] The peak O_1_ at 0.8 V is due both to direct oxidation of TPrA at gold electrode and to the formation of the Au oxide layer (Figure S5A). Indeed, the anodic growth of the surface oxides has been extensively investigated at noble metal electrodes.[^53^, ^54^] The presence of this oxide layer can have various effects on redox reactions of species in solution.[^55^, ^56^] With increasing concentration of TPrA on both Pc and roughened gold electrodes, the anodic peak corresponding to the catalytic oxidation of TPrA by incipient gold hydrous oxides at 0.3 V and direct TPrA oxidation peak at 0.8 V increased (Figures S6A and S6B). It is noteworthy that even if the peak O_1_ increased with TPrA concentration, the cathodic R_1_ peak remained constant. The wave R_1_ corresponds to the reduction of the oxide gold layer formed during the anodic scan. The current density of R_1_ wave decreased by a factor ~4.5 from −0.89 mA/cm^2^ to −0.20 mA/cm^2^ after 120 minutes of the Cu‐Fenton smoothening (Figure 2A). It shows the progressive decrease of the formation of the Au oxide layer and thus of the electrode area with the removal of asperities by longer exposure to HO^**•**^ radicals, as imaged by AFM. On one hand, one can notice the progressive disappearance of the anodic peak O_1_ with longer exposure time of gold surface to the Cu‐Fenton reagent. After 120 minutes of ex situ attack by HO^•^, the O_1_ wave almost vanished. Indeed, the amplitude of O_1_ wave drop from an initial value of 0.58 mA/cm^2^ down to 0.11 mA/cm^2^ after 120 minutes. This indicates that the TPrA oxidation occurs predominantly on the active gold sites. Their knock‐out by HO^**•**^ decreases drastically the efficiency of the direct TPrA oxidation, which is almost quantitatively suppressed. The decrease ratio of the current density is similar to the one calculated for the cathodic process R_1_ and it reflects the decrease of the gold electrode area. The second anodic wave (O_2_) corresponds to the catalytic oxidation of TPrA (equations 1–2). At these more anodic potentials, [Ru(bpy)_3_]^2+^ is oxidized and forms [Ru(bpy)_3_]^3+^. The homogeneous oxidation of TPrA occurs by a redox reaction with the electrogenerated [Ru(bpy)_3_]^3+^ species. The oxidation wave O_2_ has to be considered carefully because the O_1_ wave is located just before with a partial overlapping. By considering only O_2_ wave (i.e. subtracting O_1_ from O_2_), one can see that the corresponding current density increased with longer HO^**•**^ attack times. This behavior can be explained by the fact that more TPrA is available for the catalytic route under this condition since TPrA has not been oxidized at milder potentials where O_1_ was observed. For additional evidence, a control experiment on HO^**•**^ radical generation by Fe‐Fenton reagent treatment was carried out. Figure S7 shows similar changes in O_1_, O_2_ and R_1_ current densities and ~2‐fold enhancement in ECL intensity upon exposing the gold electrode for 20 min in the Fe‐Fenton reagent. Beyond this duration, the quenching of ECL was observed and it was overcome by using Cu‐Fenton reagent for gold surface treatment.

As already known, the ECL intensity is highly sensitive to the electrode materials and depends on the predominant mechanistic pathway, especially on the efficiency to oxidize the sacrificial coreactant.^[42]^ Bard and co‐workers proved that direct oxidation method is predominant in ECL generation from Ru(bpy)_3_^2+^/TPrA system at higher pH^[56]^ and at more positive potentials.[^41^, ^42^] Thus, any effort in increasing the TPrA oxidation reaction rate should lead to significantly enhanced ECL intensity. Figure 2B shows that the ECL appears at the oxidation potential of [Ru(bpy)_3_]^2+^ (i.e. O_2_ wave). Indeed, at a [Ru(bpy)_3_]^2+^ concentration greater than 0.1 mM, ECL is predominantly generated by the catalytic route (otherwise EC’ route). Interestingly, the ECL signal increased when the active gold sites and the asperities were removed. The asperities removal by HO^**•**^ translates into a 2.7‐fold increase in ECL intensity. This is a very unusual behavior since a larger electrode surface area result most of the time in a stronger ECL signal. Once again, this can be explained by the higher availability of the TPrA at the oxidation potential of the [Ru(bpy)_3_]^2+^. In other words, the TPrA coreactant is not consumed uselessly during O_1_ oxidation process and thus its concentration at the electrode surface remains that of the bulk solution until the potential sweep becomes sufficient to generate [Ru(bpy)_3_]^3+^ (i.e., O_2_ wave). On the surface attacked by HO^•^ generated by the ex situ Cu‐Fenton treatment, the ECL process follows predominantly the ECL catalytic route that can be described by the following reaction Scheme:^[57]^

(1)

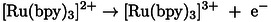



(2)

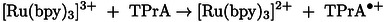



(3)

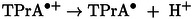



(4)

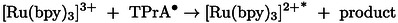



(5)

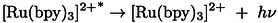




To further analyze the ECL behavior on this surface‐tuned gold electrode, we plotted the ECL peak intensity as function of the HO^**•**^ attack time (Figure 2C). A linear correlation was obtained with a R^2^ value of 0.99. This trend is also evidenced by plotting the ECL signal as function of the roughness factor (Figure 2D). For all the tested *R_RMS_*, ECL enhancement correlates with the exposure time to HO^**•**^ and the general tendency is an increase of the ECL emission with lower *R_RMS_* values. Considering the preliminary electrochemical study, it indicates that the resulting ECL signal is more influenced by the outer‐sphere [Ru(bpy)_3_]^2+^ oxidation than by the active site‐dependent direct TPrA oxidation reaction. This is coherent with the catalytic route for ECL generation.

The large number of asperities at the surface of a rough electrode favors the formation of an extensive oxide monolayer that inhibits direct TPrA oxidation and translates in a lower ECL intensity. With these asperities being knocked out by HO^**•**^, the number of active sites responsible for TPrA oxidation decreases, which again reduces the occurrence of light‐emission following a direct oxidation process. However, as the roughness or asperities on the electrode surface decreases, enhanced ECL was observed along with a drop in direct TPrA oxidation current and with improved kinetics of Ru(bpy)_3_^2+^ oxidation at more positive potentials generating more Ru(bpy)_3_^3+^ intermediates that are involved in the ECL emitting reactions (Figures S8A−B). These results revealed that a less intense ECL on a rough electrode occurs via direct oxidation process (equations S9–S13) whereas enhanced ECL on a smooth electrode was a result of the catalytic oxidation mechanism (equations 1–5).

Additional evidences on electrocatalytic deactivation by asperities elimination were gathered for roughened and smoothened gold electrodes in 0.1 M PBS in the absence or presence of 100 mM TPrA and 1 mM Ru(bpy)_3_^2+^ (Figure S5). In Figure S5B, the disappearance of the anodic peak at 0.3 V and the decrease in the current density of the peak at 0.8 V reveal that the asperities responsible for effective TPrA oxidation are removed by smoothening. In the meantime, the reduction peak current and peak potential of the outer‐sphere redox reaction of [Ru(bpy)_3_]^2+^ remain unchanged (Figure S5). The influence of TPrA and [Ru(bpy)_3_]^2+^ oxidation on the surface asperities of gold electrode were further studied (Figures S5C−D) to consolidate the role of asperities in inner‐ and outer‐sphere electrochemical reactions, respectively. All these results point out that the asperities on gold electrode surface selectively catalyze reactions involving free radical intermediates, and their removal causes the electrocatalytic deactivation of the resulting gold electrode. The usefulness of a smooth electrode surface for enhanced ECL was demonstrated by a lower background intensity,^[58]^ despite the higher current densities on a rough gold electrode as shown in Figures S6A−D. In addition, as displayed on Figures S6E−F, the lower potential oxidation of TPrA at 0.3 V has no influence on Ru(bpy)_3_^2+^ ECL and reflects solely the effect of surface asperities on the catalytic oxidation activity.

**Figure 3:**
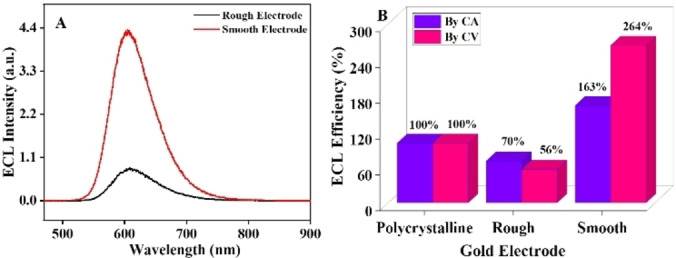
ECL spectrum (A) and ECL efficiency (B) of Pc−Au electrode measured by chronoamperometry or voltammetry in 0.1 M PBS (pH 7.4), 10 mM TPrA and 1 mM [Ru(bpy)_3_]^2+^ before and after exposure to HO^**•**^ for 120 minutes.

The ECL spectrum (Figure 3A) with a maximum at λ=608 nm corresponds to the emission from [Ru(bpy)_3_]^2+^* excited state and it matches with its photoluminescence (PL) spectrum (Figure S9). Also, the peak intensity of the ECL spectrum increased by 5 folds when comparing a rough and a smooth electrode surface. The relative ECL efficiency between a rough and a smooth gold electrode was calculated using chronoamperometry (CA) and CV (Figure S10–11). It reveals that the ECL efficiency on the rough surface is 30 % lower compared to a Pc surface when taking into account the difference in surface area whereas the smoothened electrode offers a 2.7‐fold increase as shown in Figure 3B.

**Figure 4:**
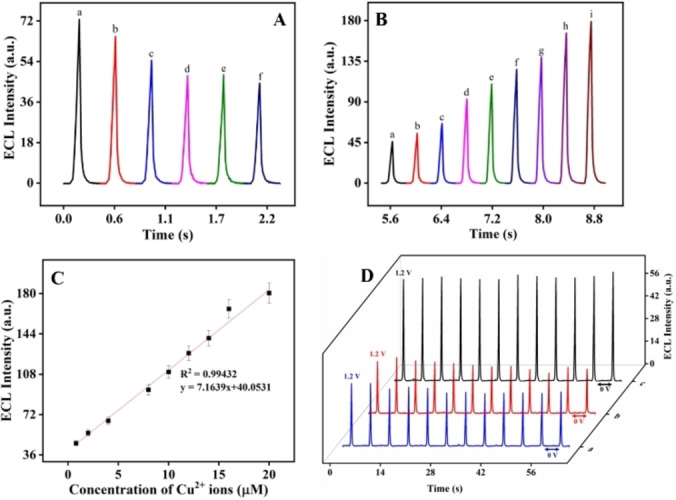
ECL peaks recorded on Pc−Au in 10 mM PBS with 1 mM TPrA and 0.1 mM [Ru(bpy)_3_]^2+^ containing 0 μM (a) 20 μM (b) 40 μM (c) 60 μM (d) 80 μM (e) 100 μM (f) of H_2_O_2_ (A) and 0.8 μM (a), 2 μM (b), 4 μM (c), 8 μM (d), 10 μM (e), 12 μM (f), 14 μM (g), 16 μM (h), 20 μM (i) of CuSO_4_ (B). Linearity plot (C) of Pc−Au in 10 mM PBS with 1 mM TPrA 0.1 mM [Ru(bpy)_3_]^2+^and 100 μM H_2_O_2_ with a varying CuSO_4_ concentration. Stability plot (D) of Pc−Au in 10 mM PBS with 1 mM TPrA and 0.1 mM [Ru(bpy)_3_]^2+^ (black), 100 μM H_2_O_2_ (red) and 20 μM CuSO_4_ (blue).

As reported above, the ex situ smoothening of the electrode surface was effective but quite time‐consuming (up to 2 hours of chemical treatment prior to recording ECL). Thus, we investigated a comparable smoothening of the roughened gold electrode following an in situ approach (Figure S12) and analyzed its action on surface modification by AFM imaging (Figure S1B). Just like the ex situ method based on Cu‐Fenton reagent, the in situ method employs H_2_O_2_ and CuSO_4_ but both chemicals were directly added to the ECL solution containing 10 mM PBS, 1 mM TPrA and 0.1 mM [Ru(bpy)_3_]^2+^. Here, the idea is to form HO^•^ radicals directly in the ECL solution by simply adding CuSO_4_ and H_2_O_2_ in the electrolyte. The Cu^2+^ cations introduced into the ECL system are solely used as a surface treatment catalyst and do not take part in the electrochemical reactions involved in the ECL mechanism. Besides the decrease in current densities of gold redox peaks as observed for the ex situ method, redox peaks at around 0.24 V and 0.12 V were observed in Figure S12A confirming the presence of remaining copper species inside the electrolyte when performing the in situ method. H_2_O_2_ is also oxidized at the anodic potential required to generate ECL (Figure S12). Both points are further discussed in the Supporting Information (section 2.7). In addition to smoothening the gold electrode surface by in situ Cu‐Fenton reaction, it is possible to detect the Cu^2+^ ions quantitatively according to their effect on the ECL signal. Initially, the presence of H_2_O_2_ lowers the ECL intensity due to its predominant quenching effect rather than smoothening of the electrode surface (Figure 4A). Then, in a Ru(bpy)_3_^2+^/TPrA solution containing 100 μM H_2_O_2_, a gradual increase in ECL intensity was observed upon addition of CuSO_4_ in the range from 0.8 to 20 μM (Figure 4B). The corresponding calibration curve is linear and exhibits a R^2^ value of 0.99 (Figure 4C). Also, the LOD value was found to be 0.75 μM. This enhanced ECL signal was found to be stable for at least 12 cycles (i.e., up to 60 s) when applying successive potential steps of 0 V for 2 s and 1.2 V for 0.3 s. The ECL signal intensity of the in situ smoothened gold electrode was found to be around 1.7 times higher than that of a rough gold electrode in presence of hydrogen peroxide, expressing the effect of asperities removal by Cu‐Fenton generated HO^•^ on ECL reaction mechanism and kinetics.

## Conclusion

In summary, the enhancement of ECL intensity signal on gold surface is achieved by selectively knocking out the Au active sites and the asperities by HO^**•**^ radicals that are generated either ex situ or in situ with Cu‐Fenton reagent. A gradual decrease in R_RMS_ value was observed together with a concomitant increase of the corresponding ECL intensity with the treatment duration. It confirms the formation of an atomically smoothened gold surface and also that the ECL reaction mechanism switched from a direct route to a catalytic route when employing the standard ECL system composed of [Ru(bpy)_3_]^2+^ and TPrA. Precisely, the completely smoothened gold surface affords a 2.7‐times higher ECL intensity, reflecting 163 % of ECL efficiency compared to a roughened gold surface. In addition, the in situ Cu‐Fenton treatment directly in the presence of [Ru(bpy)_3_]^2+^/TPrA enables to observe an initial two‐fold quenching in ECL signal by H_2_O_2_ but a significant recovery of the ECL signal in presence of CuSO_4_ that promotes the generation of HO^•^. The resulting ECL signal was stable for at least 60 s and therefore utilized for the detection of Cu^2+^ cations with a LOD value of 0.75 μM. This proof‐of‐concept investigation paves the way for not only understanding the physicochemical properties of gold metal surfaces but also opens new doors for developing more sensitive ECL‐based immunosensors and imaging strategies.

## Conflict of Interests

The authors do not declare any conflict.

1

## Supporting information

As a service to our authors and readers, this journal provides supporting information supplied by the authors. Such materials are peer reviewed and may be re‐organized for online delivery, but are not copy‐edited or typeset. Technical support issues arising from supporting information (other than missing files) should be addressed to the authors.

Supporting Information

## Data Availability

The data that support the findings of this study are available from the corresponding author upon reasonable request.
