# Supplementary material for: Enhanced Electrochemiluminescence by Knocking Out Gold Active Sites
Source: Angew Chem Int Ed Engl. 2024 Dec 27;64(10):e202421185. doi: 10.1002/anie.202421185 (PMC11878343; doi:10.1002/anie.202421185)
Supplement: Supplementary file 1 — Supporting Information [file ANIE-64-e202421185-s001.pdf]

Angewandte Chemie
Eine Zeitschrift der Gesellschaft Deutscher Chemiker

Supporting Information

Enhanced Electrochemiluminescence by Knocking Out Gold Active Sites

I. Leka Kottaiveedu Sivakumar, L. Bouffier, N. Sojic, S. Senthil Kumar**

Supporting Information
©Wiley-VCH 2021
69451 Weinheim, Germany

Enhanced Electrochemiluminescence by Knocking Out Gold Active Sites

Indhu Leka Kottaiveedu Sivakumar,[a,b] Laurent Bouffier,[c] Neso Sojic*[c] and Shanmugam Senthil Kumar*[a,b]

[a] Dr. S. Senthil Kumar and Indhu Leka K. S.
Electrodics and Electrocatalysis Division,
CSIR-Central Electrochemical Research Institute (CSIR-CECRI) Campus,
Karaikudi, Tamil Nadu 630003, India

[b] Dr. S. Senthil Kumar and Indhu Leka K. S.
Academy of Scientific and Innovation Research (AcSIR), Ghaziabad 201002, India
E-mail: ssenthilkumar@cecri.res.in

[c] Prof. N. Sojic and Dr. L. Bouffier
University of Bordeaux, CNRS, Bordeaux INP, ISM, UMR, 5255, F-33400 Talence, France
E-mail: sojic@u-bordeaux.fr

Table of Contents

1. Experimental Procedures **S2**
1.1. Chemicals S2
1.2. Electrochemical and ECL measurements S2
1.3. *Ex Situ* Smoothening of Rough Gold Electrode S2
1.4. Cu^{2+} Ion Detection S2
1.5. ECL Spectrum Measurement S2
1.6. AFM Measurements S3
2. Supplementary Results **S3**
2.1. Surface Characterization of Pc-Au by AFM S3
2.2. Effect of *Ex situ* Smoothening of Gold Electrode on Electrochemical Measurements S3
2.3. Effect of *Ex situ* Smoothening of Gold Electrode on ECL Signals S7
2.4. Reaction Mechanism behind ECL Emission S7
2.5. PL Spectrum S8
2.6. ECL Quantum Efficiency Calculation S8
2.7. Effect of *in situ* Smoothening of Gold Electrode on ECL Signals S9
2.8. Limit of Detection (LOD) Calculation S11
3. References **S11**

1. Experimental Procedures

1.1. Chemicals

Tris-(2,2'-bipyridine)ruthenium(II)chloride ($[(Ru(bpy)_3]Cl_2$) and tri-*n*-propylamine (TPrA) were from Sigma Aldrich, Potassium dihydrogen phosphate (KH_2PO_4), hydrogen peroxide (H_2O_2 – 30%), potassium ferricyanide ($K_3[Fe(CN)_6]$) and sodium acetate were from Merck. Acetic acid, perchloric acid and ethylene diamine tetra acetic acid di-sodium salt (EDTA) were from Rankem. Hydroquinone and copper sulphate pentahydrate($CuSO_4.5H_2O$) were from Himedia. Sodium hydroxide (NaOH) and ferrous ammonium sulphate $(NH_4)_2Fe(SO_4)_2.6H_2O$ were from SRL. Sulphuric acid (H_2SO_4) was from Labfine Chem. Phosphoric acid (H_3PO_4) was from Emparta ACS. Sodium perchlorate monohydrate ($NaClO_4.H_2O$) was from LOBA CHEMIE and potassium chloride (KCl) was from CDH. 0.1 M PBS solution was prepared with the required amounts of KH_2PO_4 and NaOH and its pH was corrected to 7.4 using concentrated NaOH and H_3PO_4 solutions. All these chemicals were used without any purifications and all the solutions were prepared using milli-Q water.

1.2. Electrochemical and ECL Measurements

Cyclic Voltammetry (CV), Chronoamperometry (CA) and Electron Impedance Spectroscopy (EIS) measurements were recorded using a potentiostat (Autolab, Ecochemie, The Netherlands). The ECL signals were simultaneously measured along with CV curves using a photomultiplier tube (PMT-Hamamatsu H9305-04). The signal from the PMT was amplified using a high voltage power supplied amplifier held at -500V. An electrometer system connected to Autolab *via* an analog-to-digital convertor (ADC) was used to convert photocurrent generated at PMT into voltage. All the electrochemical measurements were performed using commercially available gold disc electrode of 0.07065 cm^2 surface area as working electrode, Ag/AgCl (1M KCl) and platinum foil as reference electrode and counter-electrode, respectively. Prior to any measurements, gold disc electrode was mechanically polished with 0.05 µm alumina powder on a wet polishing pad, washed and ultrasonicated for 1 minute in milli-Q water to remove any alumina powder adsorbed over the electrode surface. The electrode was then cycled between 0 V and +1.6 V (*vs* Ag/AgCl) in 0.5 M H_2SO_4 until the stable voltammogram of typically clean polycrystalline gold (Pc-Au) electrode surface was obtained. Then the clean Pc-Au gold electrode was roughened chronoamperometrically by altering the electrode potential from $E_{initial}$ = 0 V to E_{high} = +2 V (Ag/AgCl) back and forth for 750 cycles with each pulse of 20 ms long and with no quite time.[1]

1.3. *Ex Situ* Smoothening of Rough Gold electrode

The roughened electrode was washed with water and exposed to freshly prepared Cu-Fenton reagent containing 10 mM $CuSO_4$:100 mM H_2O_2 for 10 minutes and this was repeated for 120 minutes with replacing the Cu-Fenton reagent after every 10-minute interval. The reaction of Cu-Fenton reagent on the electrode was terminated by removing the electrode from the reagent and washing it with milli-Q water. Similar procedure was followed in case of Fenton's reagent of composition $(NH_4)_2Fe(SO_4)_2$ (1mM), EDTA (1 mM), acetate buffer (pH = 4.7, 10 mM) and H_2O_2 (10 mM). All the experiments were carried out under air saturated conditions.

1.4. Cu^{2+} Ion Detection

Chronoamperometric detection of Cu^{2+} ions were achieved by applying 0.6 V for 0.5 s and 1.2 V for 0.1 s to the rough gold electrode in electrolyte solution containing 10 mM PBS (pH = 7.4), 1 mM TPrA, 0.1 mM $Ru(bpy)_3^{2+}$ and 100 µM H_2O_2. To this electrolyte, $CuSO_4$ solution was added to attain electrode smoothening along with Cu^{2+} sensing.

1.5. ECL Spectrum Measurements

ECL spectrum was measured using a fluorescence spectrophotometer (Hitachi, F-7000) coupled with a potentiostat by applying a constant potential of 1.2 V for 30 s under air saturated conditions. ECL spectrum was recorded with an optimized PMT voltage as 700 V with 5 nm slit width. The gold foil of 1 × 1 cm^2 surface area was used as working

electrode, Ag/AgCl and Ag wire were used as reference and counter electrode, respectively. A quartz cuvette (3.5 ml) with 10 mm path length was used as an electrochemical cell.

1.6. AFM Measurements

Atomic Force Microscopy (AFM) images of rough and smooth gold disk electrode of 0.031 cm^2 surface area were measured under ambient conditions using a Scanning Probe Microscope (5500 series, Agilent Technologies, USA) in contact mode at scanning speed of 0.8 ln/s. The topographic and 3D images were treated in GYWDDION version 2.63 and their roughness parameters were analysed with Pico View 1.14.1 software.

2. Supplementary Results

2.1. Surface characterization of Pc-Au by AFM

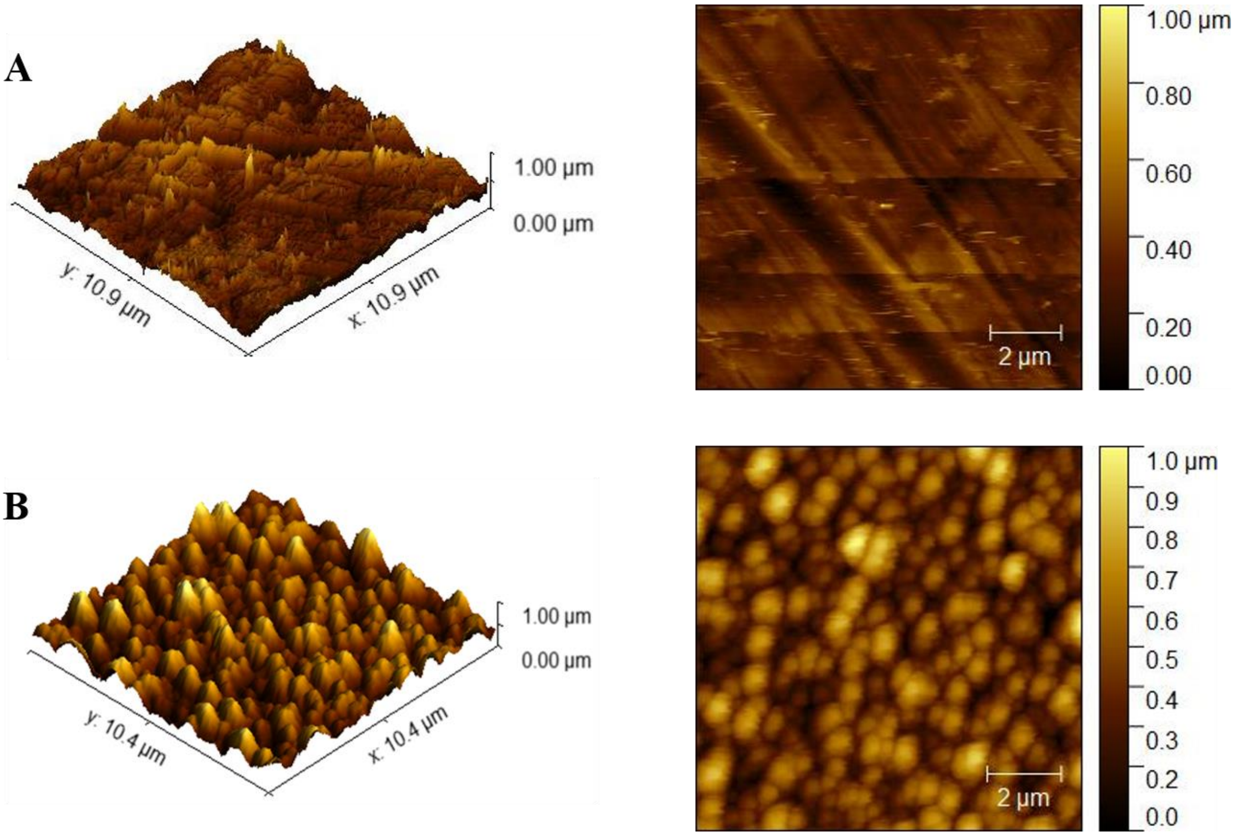

Figure S1. AFM images of polycrystalline gold electrode (A), and smooth gold electrode surface after *in situ* Cu-Fenton treatment by the ECL system containing 0.1 M PBS, 10 mM TPrA, 1 mM $Ru(bpy)_3^{2+}$, 1 mM H_2O_2 and 500 µM $CuSO_4$ solution(B) with R_{rms} value of 60.9 nm.

2.2. Effect of *Ex Situ* Smoothening of Gold Electrode on Electrochemical Measurements

The effect of Cu-Fenton treatment on selective removal of surface asperities and further electrocatalytic deactivation of gold electrode was primarily studied with cyclic voltammogram in 0.1 M H_2SO_4 (Figure S2). This

smoothening of rough gold electrode surface was accomplished by treating it with Cu-Fenton reagent containing 1:10 of Cu^{2+}:H_2O_2 composition for 120 minutes with replacing the freshly prepared reagent for every 10-minute interval.

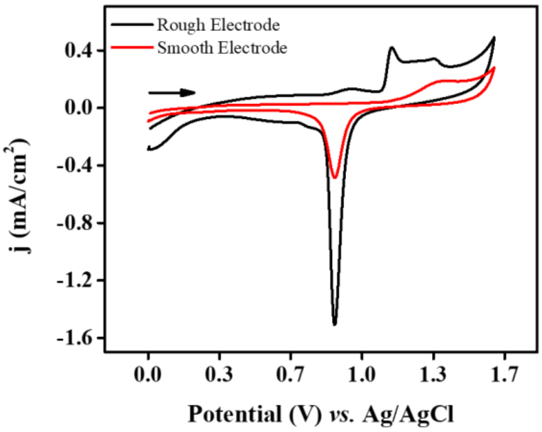

Figure S2. Cyclic voltammogram of 0.1 M H_2SO_4 before and after exposure of Au electrode to $HO^{\bullet}$ for 120 minutes.

The major reactions occurring in Cu-Fenton reagent of used composition for hydroxyl radical generation involves the following steps of advanced oxidation processes:

$$Cu^{2+} + H_2O_2 \rightarrow CuOOH^{+} + H^{+} \qquad \textbf{(S1)}$$

$$CuOOH^{+} \rightarrow Cu^{+} + H^{+} + O_2^{-} \qquad \textbf{(S2)}$$

$$Cu^{+} + H_2O_2 \rightarrow Cu^{2+} + OH^{-} + HO^{\cdot} \qquad \textbf{(S3)}$$

$$H_2O_2 + HO^{\cdot} \rightarrow H_2O + HO_2^{\cdot} \qquad \textbf{(S4)}$$

$$Cu^{2+} + HO_2^{\cdot} \rightarrow Cu^{+} + O_2 + H^{+} \qquad \textbf{(S5)}$$

At first, Cu^{2+} ions undergo complexation with H_2O_2 (S1) to form copper(II) hydroperoxide species, which immediately suffer the unimolecular decomposition as shown in equation S2 to form Cu^{+} and superoxide ion. Formed Cu^{+} ion then reacts with H_2O_2 to give hydroxyl free radicals. The excess hydrogen peroxide present is then reduced by some hydroxyl radicals to generate superoxide ions which re-reduces Cu^{2+} to Cu^{+} ions to continue the $HO^{\bullet}$ production until all hydrogen peroxide is consumed.[2][3] A comparatively sharp peak at +1.12 V in Figure S2 (black curve) shows the predominant formation gold crystal planes of Au(100) orientation over stable Au(111) and A(110)[4] due to continuous gold oxide formation/reduction and gold dissolution/redeposition during the roughening process. A small hump at 0.93 V prior to monolayer oxide formation corresponds to the premonolayer oxidation of active adatoms on electrode surface. Also, the cathodic current in the region of 0 V to +0.3 V during anodic and cathodic sweeps and charges in double layer region indicated surface oxides and other adsorbed anions (SO_4^{2-}, HSO_4^{-}) formed during electrode roughening[5] Along with the gold oxide reduction peak at 0.85 V, a shoulder peak observed at 0.73 V is probably related to surface defects.[6] Disappearance of all these anomalous peaks, in cyclic voltammogram of smooth electrode in Figure S2 (red) makes it obvious that $HO^{\bullet}$ attack on gold electrode resulted in removal of surface defects and asperities. In addition, a single anodic peak at 1.34 V with decreased peak current density from -1.51 mA/cm^2 to -0.49 mA/cm^2 clearly exhibits the smooth gold electrode surface with stable Au(111) planes present after Cu-Fenton treatment. For further understanding of Cu-Fenton action on gold electrode, Electrochemically active surface area (ECSA) of rough and smooth electrode was calculated by normalizing the gold oxide reduction charge with ($Q_{theoretical}$=) 390 µC/cm^2. The faradaic charge of gold oxide reduction was obtained by integrating the area under the reduction peak and was used for ECSA and corresponding electrode surface roughness calculation by following formulae respectively,[7]

$$\text{Charge } (Q_{real}) = \frac{\text{Integrated area under reduction peak}}{\text{Scan rate of CV}} \qquad \textbf{(S6)}$$

$$\text{ECSA} = \frac{Q_{real}}{Q_{theooretical}} \quad \textbf{(S7)}$$

$$\text{Roughness factor } (\rho) = \frac{\text{ECSA}}{\text{Geometrical Area}} \quad \textbf{(S8)}$$

The calculations revealed that the ECSA of rough and smooth electrodes were 0.155 cm^2 and 0.077 cm^2 with their corresponding roughness factor being 2.2 and 1.09, respectively. This two-fold decrease in ECSA and ρ values of gold electrode on treating with Cu-Fenton reagent complements the topographical observations of AFM images of rough and smooth electrodes, where their roughness parameters expressed decrease in values.

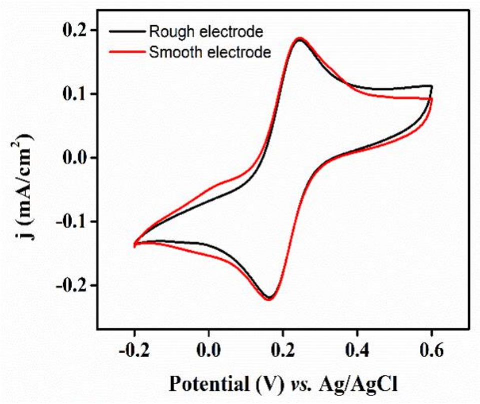

Figure S3. Cyclic voltammogram of 0.1 M KCl and 0.001 M $K_3[Fe(CN)_6]$ before and after exposure of Au electrode to $HO^•$ for 60 minutes.

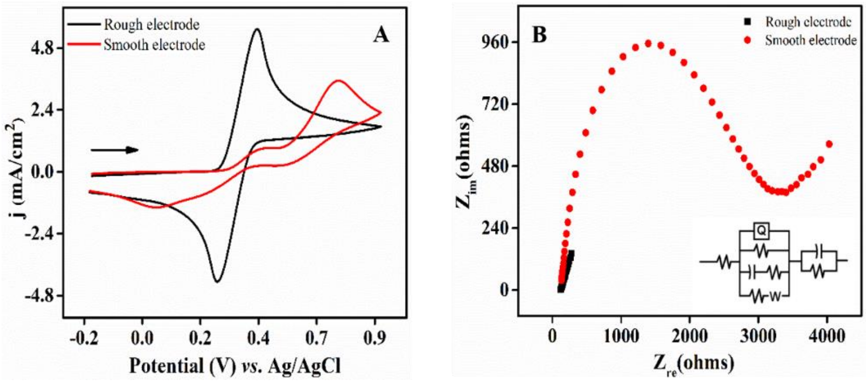

Figure S4. CV(A) and EIS(B) plots of 10 mM hydroquinone in 0.1 M $NaClO_4$ and 0.01 M $HClO_4$ before and after exposure of Au electrode to $HO^•$ for 120 minutes.

The consequences of asperities removal by Cu-Fenton reagent on kinetic parameters were examined using EIS measurements of Q/HQ system by applying small sinusoidal voltage of 0.01 V amplitude around $E_{1/2}$ value over a frequency range of 0.1 Hz to 10 kHz. The results of EIS measurements are commonly presented as Nyquist plots. Figure S4B shows the Nyquist plot of Q/HQ system consisting real (Z') and imaginary (Z") electrode impedance values. The semicircle in Figure S4(B) gives us the information about the electron transfer kinetics through charge transfer resistance (R_{CT}) value and the linear line or Warburg impedance in Figure S4B is connected to the diffusion process at electrode/electrolyte interface of Q/HQ redox system.[7] The R_{CT} values of rough and smooth gold electrode were determined by convergent fitting of Nyquist plot from EIS measurements to an equivalent circuit of R(QR(CR)(RW))(CR). As shown in Figure S4B, the bigger semicircle of smooth gold electrode compared to rough gold electrode indicates the enhanced charge transfer resistance and depleted electrical conductivity of surface deactivated gold electrode. This verifies how the $HO^•$ radicals generated from Cu—Fenton removes asperities on rough gold electrode, which had higher

conductivity, low R_{CT} of 207.5 Ω and fast electron transfer kinetics to lead a reversible Q/HQ redox reaction and resulting in smooth gold electrode with lower conductivity, high R_{CT} of 745.9 Ω and slow irreversible electron transfer kinetics of Q/HQ redox reactions.

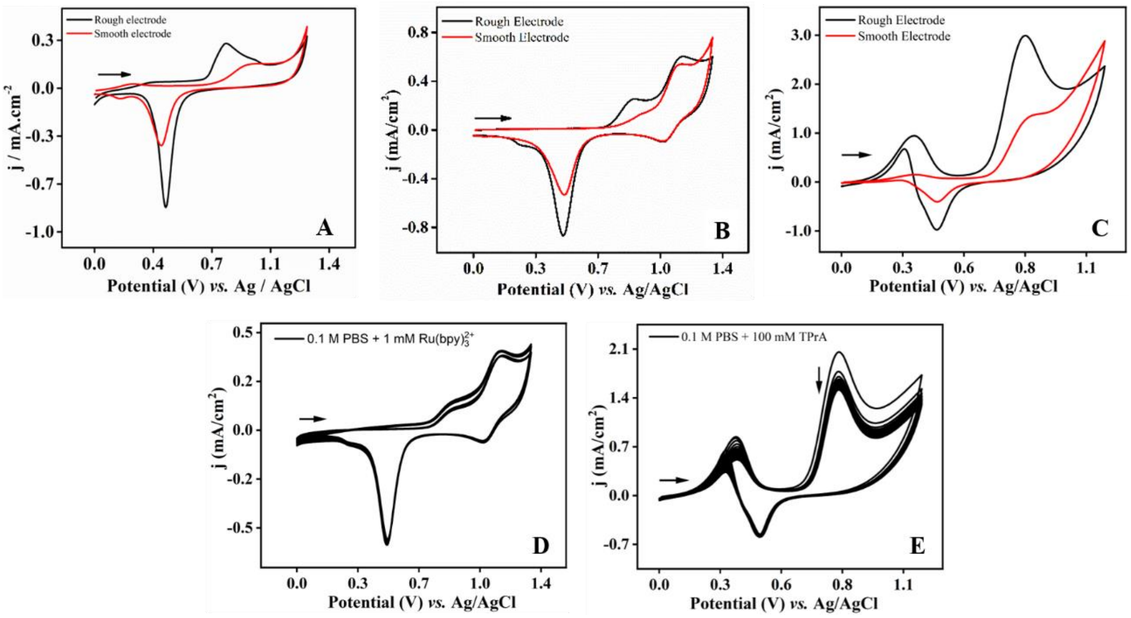

Figure S5. Cyclic Voltammogram of rough Au electrode (black line, before Cu-Fenton treatment for 120 minutes in 0.1 M PBS (pH = 7.4) and smooth red line, i.e. after treatment) without (A) or with 1 mM Ru[(bpy)$_3$]$^{2+}$ (B) or 100 mM TPrA (C) at 0.1 V/s scan rate. Rough gold electrode in 0.1 M PBS containing 1 mM [Ru(bpy)$_3$]$^{2+}$ (D) or 100 mM TPrA (E) swept between 0 to 1.3 V at 50 mV/s for 20 cycles.

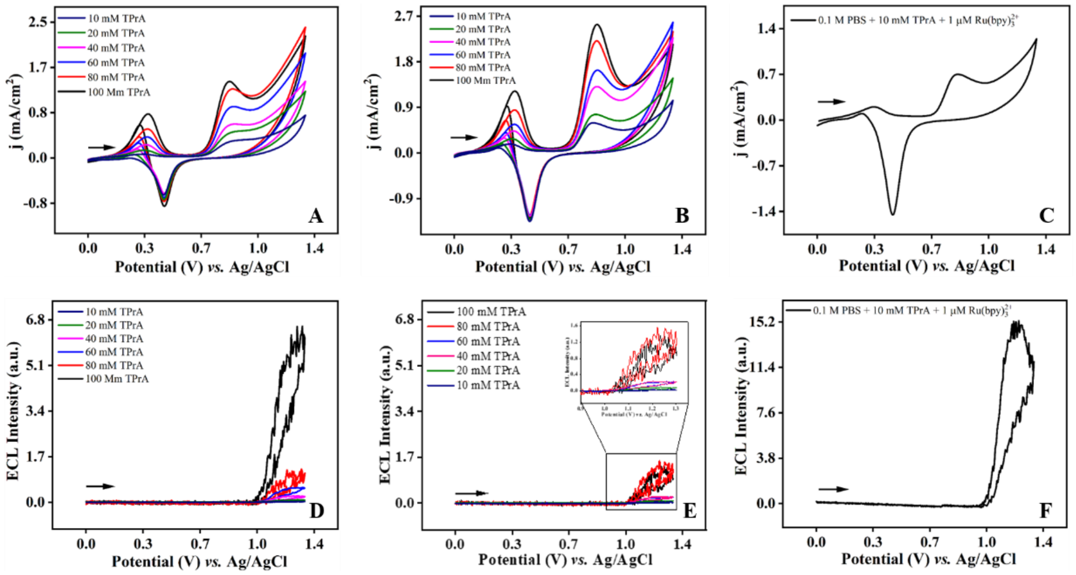

Figure S6. CV and ECL plots of Polycrystalline (A,D) and Rough (B,E) gold electrode respectively, in 0.1 M PBS and TPrA solution at varying concentration (Inset of E: Zoomed plot of rough gold electrode in 0.1 M PBS and TPrA), CV (C) and ECL (F) of rough gold electrode in 0.1 M PBS (pH = 7.4), 10 mM TPrA and 1 μM Ru(bpy)$_3^{2+}$ with potential scanned from 0 V to 1.3 V at 0.1 V/s scan rate.

2.3. Effect of *Ex Situ* Smoothening of Gold Electrode on ECL Signals

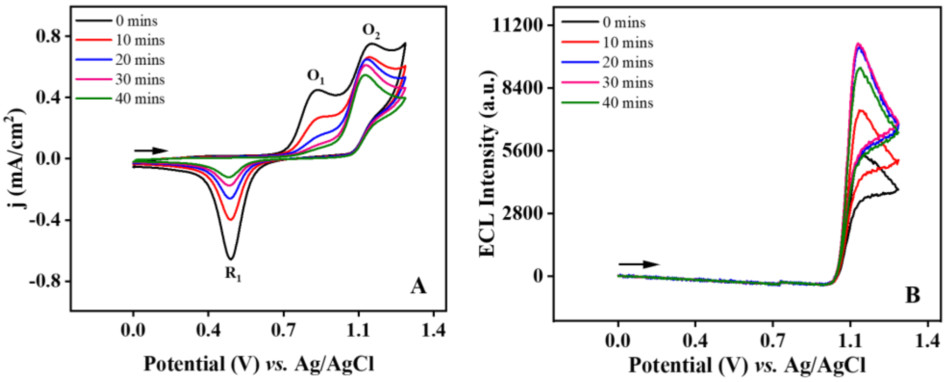

Figure S7. CV(A) and ECL(B) plots of 10 mM TPrA and 1 mM $Ru(bpy)_3^{2+}$ in 0.1 M PBS (pH = 7.4) before and after exposure of Au electrode to $HO^{\bullet}$ by Fe-Fenton reagent.

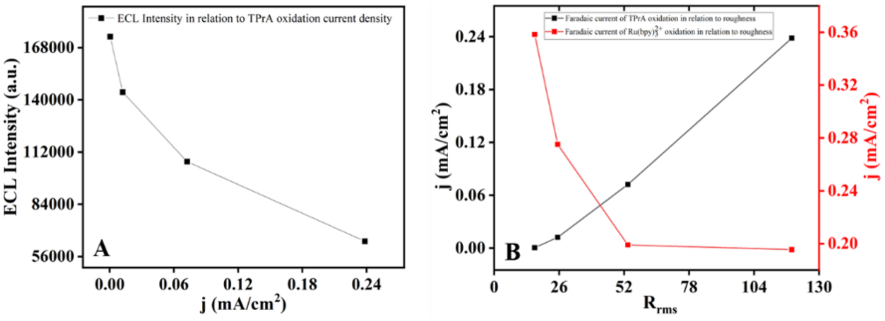

Figure S8. Plot relating ECL Intensity with to TPrA oxidation current density (A). Plot relating surface roughness factor (R_{rms}) to TPrA and $[Ru(bpy)_3]^{2+}$ oxidation current density (B).

2.4. Reaction mechanism behind ECL emission

The above observations of suppressed TPrA oxidation and enhanced $Ru(bpy)_3^{2+}$ oxidation in smooth electrode exhibiting elevated ECL emission can be explained by alteration in emission mechanism on rough and smooth gold electrodes. The less intense ECL signal of $Ru(bpy)_3^{2+}$/TPrA system in rough electrode was due to direct oxidation method (Scheme S1), where both TPrA and $Ru(bpy)_3^{2+}$ directly oxidize on electrode surface to provide reactive intermediates that interacts with each other to cause light emission.

$$\mathrm{Ru(bpy)_3^{2+} - e^- \rightarrow Ru(bpy)_3^{3+}} \qquad \textbf{(S9)}$$

$$\mathrm{TPrA - e^- \rightarrow TPrA^{\cdot +}} \qquad \textbf{(S10)}$$

$$\mathrm{TPrA^{\cdot +} \rightarrow TPrA^{\cdot} + H^+} \qquad \textbf{(S11)}$$

$$\mathrm{Ru(bpy)_3^{3+} + TPrA^{\cdot} \rightarrow Ru(bpy)_3^{2+*} + Pr_2N^+ = CHEt} \qquad \textbf{(S12)}$$

$$\mathrm{Ru(bpy)_3^{2+*} \rightarrow Ru(bpy)_3^{2+}} + h\nu \qquad \textbf{(S13)}$$

SCHEME S1. Direct oxidation route of generation of ECL signals

ECL signal with enhanced intensity in smooth electrode after Cu-Fenton treatment for 120 minutes was a result of catalytic oxidation mechanism, which involves the homogeneous oxidation of TPrA by reaction with electrogenerated

$Ru(bpy)_3^{3+}$ to generate highly reductive radical and further interactions of $Ru(bpy)_3^{3+}$ and $TPrA^{\bullet}$ for luminescence emission. In either mechanism, the production of ECL signals were due to $Ru(bpy)_3^{2+*}$ excited species.

2.5. PL Spectrum

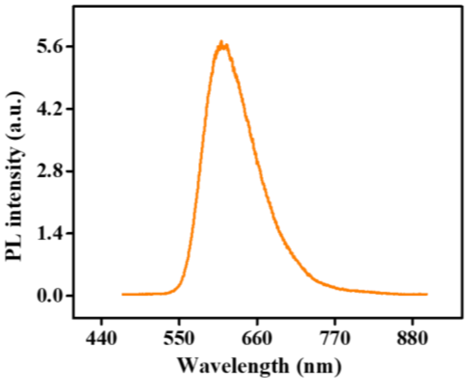

Figure S9. PL spectrum of 1 mM $Ru(bpy)_3^{2+}$ in 0.1 M PBS (pH=7.4) and 10 mM TPrA

2.6. ECL Quantum efficiency calculation

ECL quantum yield or efficiency is a significant parameter that determines the performance of an ECL system. Primarily, ECL efficiency (Φ_{ECL}) is defined as the ratio of number of photons emitted to the faradaic charges passed during the ECL experiments. Here, we calculated relative ECL efficiency using the formula,[8][9]

$$\Phi_{ECL} = \frac{\left[\int \frac{\text{ECL dt}}{\text{Current dt}}\right]x}{\left[\int \frac{\text{ECL dt}}{\text{Current dt}}\right]st} \times \Phi^{\circ}_{ECL} = \frac{\left[\int \frac{\text{ECL dt}}{\text{Current dt}}\right]x}{\left[\int \frac{\text{ECL dt}}{\text{Current dt}}\right]st} \times 100\% \qquad \text{(S14)}$$

Where Φ°_{ECL} is the ECL efficiency of standard luminophore $Ru(bpy)_3^{2+}$ and is considered as 100%. Electrochemical current and ECL intensity values represented as current and ECL in the above formula were used to calculate the ECL efficiency of rough and smooth gold electrode separately in $Ru(bpy)_3^{2+}$/TPrA system which are stated as x. $Ru(bpy)_3^{2+}$/TPrA system in clean polycrystalline electrode was served as the reference and was referred as st.

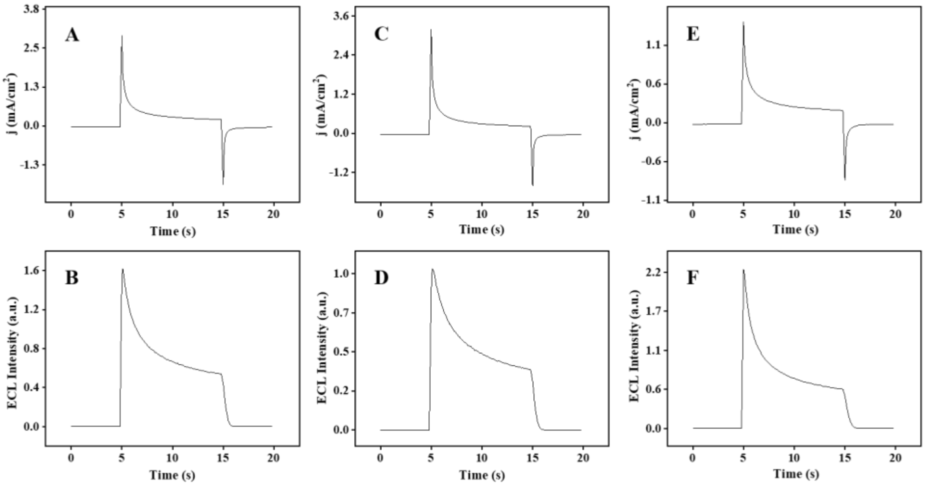

Figure S10. Chronoamperometric curves of 10 mM TPrA, 1 mM $Ru(bpy)_3^{2+}$ and 0.1 M PBS (pH = 7.4) in Polycrystalline(A), Rough(B) and Smooth(C) gold electrode and their corresponding ECL curves (B, D, F).

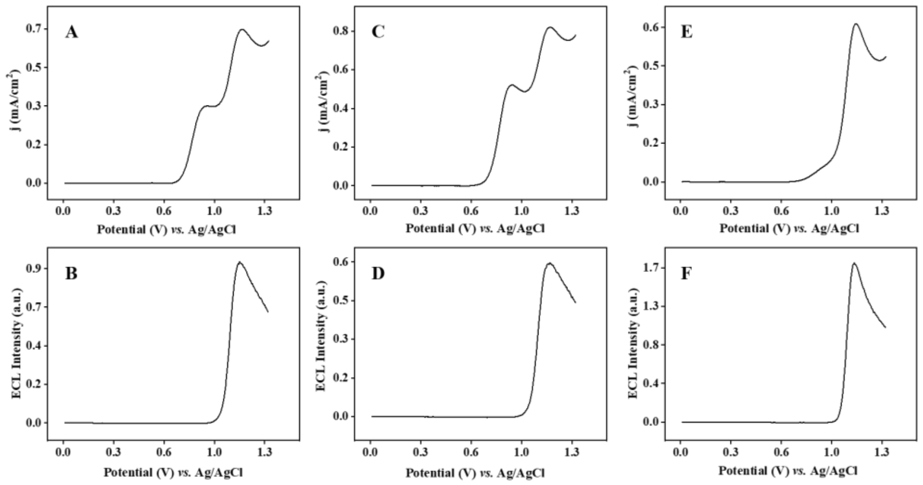

Figure S11. Voltammetric curves of 10 mM TPrA, 1 mM $[Ru(bpy)_3]^{2+}$ and 0.1 M PBS (pH = 7.4) in polycrystalline (A), rough (C) and smooth (E) gold electrode and their corresponding ECL curves (B, D, F) respectively.

Rough gold electrode,

From CA: $$\Phi_{\mathrm{ECL}} = \left(\frac{5.4069}{3.54288E{-}4}\right) \times \left(\frac{3.58951E{-}4}{7.83482}\right) \times 100\% = 70\%$$

From CV: $$\Phi_{\mathrm{ECL}} = \left(\frac{0.14725}{19.7438E{-}5}\right) \times \left(\frac{15.2547E{-}5}{0.20383}\right) \times 100\% = 56\%$$

Smooth gold electrode,

From CA: $$\Phi_{\mathrm{ECL}} = \left(\frac{8.72549}{2.44822E{-}4}\right) \times \left(\frac{3.58951E{-}4}{7.83482}\right) \times 100\% = 163\%$$

From CV: $$\Phi_{\mathrm{ECL}} = \left(\frac{0.35226}{9.97183E{-}5}\right) \times \left(\frac{15.2547E{-}5}{0.20383}\right) \times 100\% = 264\%$$

Chronoamperometric experiments were used to obtain corresponding ECL measurements by applying 1.13 V potential for 10 s. The chronoamperometric current curves and ECL intensity curves in Figure S10 were integrated and the resulting values were substituted in equation (S14) to calculate Φ_{ECL} values of rough and smooth gold electrodes. It is clear from the Φ_{ECL} values that rough electrode with more asperities on its surface than polycrystalline gold electrode, provides lesser efficient ECL signal of 70% efficiency and smooth electrodes expressed prominently elevated efficiency of ECL signal with 163% that of standard system as the amount of asperities on the surface were dropped after Cu-Fenton treatment. This observation is complemented by the efficiency values calculated from cyclic voltammetric measurements (Figure S11), where the faradaic current of forward scan and their corresponding ECL curves were integrated to determine the Φ_{ECL} values by equation (S14).

2.7. Effect of *in situ* Smoothening of Gold Electrode on ECL Signals

Previously, Raju et al.[10] developed a turn-on sensor for Cu^{2+} ions based on Cu-Fenton chemistry. They utilized Cu^{2+} ion to chemically catalyze H_2O_2 reduction to $HO^{\bullet}$ radicals which takes place in reaction with electrogenerated P-CQDs$^{\bullet -}$ to emit ECL. Here, the Cu^{2+} ions act purely as chemical catalyst without any role in electrochemical reactions.

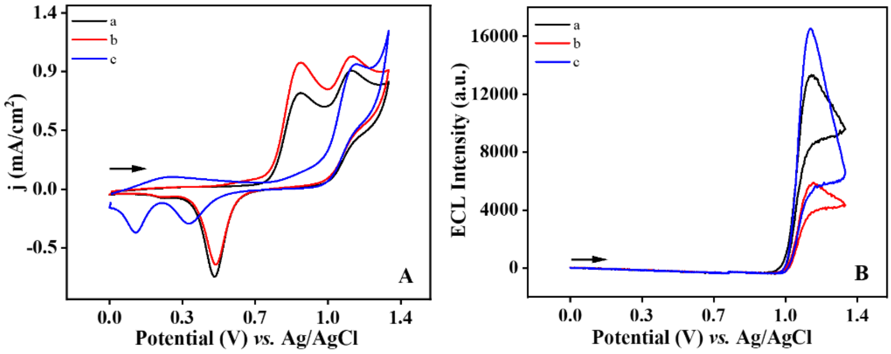

Figure S12. Voltammetric (A) and ECL (B) plots of Pc-Au in 0.1 M PBS (pH = 7.4) with 10 mM TPrA and 1 mM $Ru(bpy)_3^{2+}$(a) containing 1 mM H_2O_2 (b) and 500 mM $CuSO_4$ (c) solution at 100 mV/s.

In light of the aforementioned work, we tried smoothening the gold electrode in an *in situ* manner by introducing H_2O_2 and $CuSO_4$ to the $Ru(bpy)_3^{2+}$/TPrA ECL system. Figure S12 shows the cyclic voltammogram (A) and corresponding ECL signal (B) of gold electrode, which was roughened by the procedure mentioned in the experimental section. All the experimental parameters were identical as that of the measurements during the *ex situ* smoothening of gold electrode. In Figure S12A (black curve), anodic peaks at 0.89 V and 1.12 V indicated the monolayer oxidation of gold electrode, oxidation of TPrA, and $Ru(bpy)_3^{2+}$ and the cathodic peak at 0.48 V indicated the reduction of gold α-oxides on the electrode. When 1 mM H_2O_2 was added to the system, a slight decrease in reduction current density from -0.67 mA/cm^2 to -0.58 mA/cm^2, increase in current densities of anodic peaks of TPrA, H_2O_2 and $Ru(bpy)_3^{2+}$ oxidation was observed, which displays slight smoothening of gold electrode and small negative shift in TPrA oxidation potential was seen. Despite of the better kinetics of direct oxidation of $Ru(bpy)_3^{2+}$/TPrA system found from Figure S7A (red curve), the ECL signal in Figure S12B (red curve) exhibited 2.3-fold depletion in emission intensity. This observation can be regarded by the quenching effect of H_2O_2 on the ECL system which predominates the selective knockout of asperities by little quantity of $^{\bullet}OH$ radicals generated from H_2O_2 degradation.[11] Subsequent addition of $CuSO_4$ to the $Ru(bpy)_3^{2+}$/TPrA system led to the consecutive increase in ECL intensity (Figure S12B, blue curve). This observed elevation was correlated with the changes in the peak potential and current density shown in Figure S12A (blue curve). Along with $CuSO_4$ addition, current density of gold monolayer oxidation peak at 0.89 V decreases and a slight negative shift in oxidation onset potential of $Ru(bpy)_3^{2+}$ with no change in its redox peak current densities were observed. Above all, a negative shift in gold reduction peak potential from 0.49 V to 0.37 V coupled with decrease in its current density revealed the elimination of asperities from gold electrode surface. These variations in CV curves accompanied by the ECL signal enhancement were a combined result of two different process that involves, Cu-Fenton reagent action where Cu^{2+} ions chemically convert H_2O_2 to $HO^{\bullet}$ radicals which removes asperities and smoothens the electrode surface, increasing $Ru(bpy)_3^{2+}$ oxidation kinetics that encourages higher emission of ECL signals by catalytic oxidation route. In the second way, $CuSO_4$ added to the ECL system consumed H_2O_2 which were available for annihilation with $TPrA^{\bullet}$ so more number of TPrA reactive species will react with $Ru(bpy)_3^{3+}$ to generate luminescence.

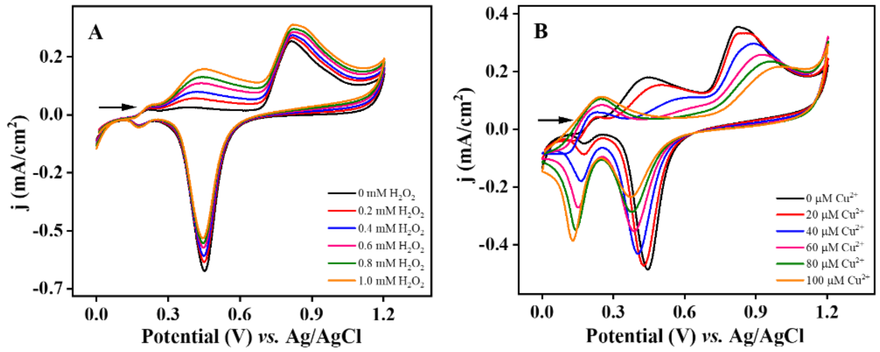

Figure S13. CV plots of Pc-Au in 0.1 M PBS (pH = 7.4) with H_2O_2 (A) and H_2O_2 + $CuSO_4$ (B).

To gain better understanding of *in situ* action of Cu-Fenton reagent in ECL system, control experiments were performed by cyclic voltammetry of rough gold electrode in 0.1 M PBS (pH = 7.4) with H_2O_2 followed by $CuSO_4$ addition to the electrolyte solution. It was observed from Figure S13A that H_2O_2 addition did not cause any significant change on gold electrode surface as no drastic changes in gold redox peaks were seen. Anodic peak around 0.42 V with increasing current densities on successive addition of H_2O_2 indicates the electrochemical oxidation of H_2O_2 to O_2[12] and is confirmed that mere H_2O_2 presence does not involve in removal of gold surface asperities. The peak shifts in cyclic voltammogram of rough gold electrode in 0.1 M PBS and 1 mM H_2O_2 with consecutive addition of $CuSO_4$ solution were displayed in Figure S13B. The redox peaks at E_a = 0.24 V and E_c = 0.1 V increased in current density with each 20 μM $CuSO_4$ addition indicating the Cu^{1+}/Cu^{2+} redox reaction occurrence as we know that Cu^{2+} to Cu^0 redox reaction in non-complexing medium follows two steps including Cu^{1+} formation prior to Cu^0 deposition.[13] The Cu-Fenton reagent action was corroborated by depletion in current density of H_2O_2 oxidation peak in Figure S13B, which stated the utilization of H_2O_2 by Cu^{2+} ions in hydroxyl radical generation to smoothen and deactivate the gold electrode surface via asperities eradication. The consequence of this Cu-Fenton action was revealed by receding in gold oxide redox current densities and by deviation in their redox potential towards irreversibility of redox reaction. The *in situ* Cu-Fenton treatment of gold electrode depletes the amount of asperities readily available for oxidation thus lesser ECSA for Au_2O_3 formation was available on the deactivated gold electrode surface when more positive potential was applied. This reflected over the current density of gold oxide reduction peaks with negative shifts in the reduction peak potentials due to the stability of Au_2O_3 formed on the deactivated gold electrode surface by hydroxyl radical attacks.[14][15]

2.8. Limit of Detection (LOD) Calculation

When $CuSO_4$ solution was added to the $Ru(bpy)_3^{2+}$/TPrA system containing 100 μM of H_2O_2 in it, a gradual increase in ECL signal intensity was noted in the range of 0.8 to 20 μM $CuSO_4$ concentration. The excellent linearity in the increase in ECL intensity was observed with increasing $CuSO_4$ solution concentration and the linearity plot (Figure 4C) was used to calculate the limit of detection of Cu^{2+} ions using the formula,

$$\text{LOD} = \frac{3 \times \text{standard deviation}}{\text{slope}} \qquad \textbf{(S15)}$$

Which turned out to be 0.75 μM. Though the linearity range for Cu^{2+} detection is less, this experiment opens the door for new development of simple and efficient sensors in future.

3. References

[1] J. Zhang, Z. Kuang, H. Li, S. Li, F. Xia, *J. Electroanal. Chem.* **2022**, *919*, 116541.
[2] J. F. Perez-Benito, *J. Inorg. Biochem.* **2004**, *98*, 430–438.
[3] Z. Jiao, H. Gong, Y. Peng, G. Zhou, X. Zhang, X. Gao, Y. Liu, *Environ. Eng. Res.* **2022**, *27*, 210305.
[4] C. Jeyabharathi, P. Ahrens, U. Hasse, F. Scholz, *J. Solid State Electrochem.* **2016**, *20*, 3025–3031.
[5] L. D. Burke, A. P. O'Mullane, *J. Solid State Electrochem.* **2000**, *4*, 285–297.
[6] Y. Xu, *Int. J. Hydrogen Energy* **2009**, *34*, 77–83.
[7] N. D. Zakaria, M. H. Omar, N. N. Ahmad Kamal, K. Abdul Razak, T. Sönmez, V. Balakrishnan, H. H. Hamzah, *ACS Omega* **2021**, *6*, 24419–24431.
[8] A. Fiorani, V. Eßmann, C. S. Santos, W. Schuhmann, *ChemElectroChem* **2020**, *7*, 1256–1260.
[9] B. Zhao, Y. Luo, X. Qu, Q. Hu, J. Zou, Y. He, Z. Liu, Y. Zhang, Y. Bao, W. Wang, L. Niu, *J. Phys. Chem. Lett.* **2021**, *12*, 11191–11198.
[10] C. Venkateswara, G. Kalaiyarasan, S. Paramasivam, J. Joseph, S. Senthil, *Electrochim. Acta* **2020**, *331*, 135391.
[11] J. Zhao, Y. He, K. Tan, J. Yang, S. Chen, R. Yuan, *Anal. Chem.* **2021**, *93*, 12400–12408.
[12] M. Gerlache, Z. Senturk, G. Quarin, J. M. Kauffmann, *Electroanalysis* **1997**, *9*, 1088–1092.
[13] X. Zeng, S. Bruckenstein, *J. Electroanal. Chem.* **1999**, *461*, 131–142.
[14] S. Cherevko, A. A. Topalov, A. R. Zeradjanin, I. Katsounaros, K. J. J. Mayrhofer, *RSC Adv.* **2013**, *3*, 16516–16527.
[15] S. Cherevko, A. R. Zeradjanin, A. A. Topalov, G. P. Keeley, K. J. J. Mayrhofer, *J. Electrochem. Soc.* **2014**, *161*, H501–H507.
